# Real-world cost analysis of chemotherapy for colorectal cancer in Japan: detailed costs of various regimens during the entire course of chemotherapy

**DOI:** 10.1186/s12913-015-1253-x

**Published:** 2016-01-04

**Authors:** Shuichi Yajima, Hisanori Shimizu, Hiroyuki Sakamaki, Shunya Ikeda, Naoki Ikegami, Jun-Ichiro Murayama

**Affiliations:** 1 Department of Health Policy and Management, School of Medicine, Keio University, 35, Shinanomachi, Shinjuku-ku, Tokyo 160-8582 Japan; 2 Taiho Pharmaceutical Co., Ltd., 1-27, Kandanishiki-cho, Chiyoda-ku, Tokyo 101-8444 Japan; 3 Department of Pharmacy Services, Showa University Hospital, 1-5-8, Hatanodai, Shinagawa-ku, Tokyo 142-8666 Japan; 4 School of Management, Tokyo University of Science, 500, Shimokiyoku, Kuki, Saitama 346-8512 Japan; 5 Department of Pharmaceutical Sciences, School of Pharmacy, International University of Health and Welfare, 2600-1 Kitakanemaru, Otawara, Tochigi 324-8501 Japan; 6 Keio University, 5-29-20-409 Shiba, Minato-ku, Tokyo 108-0014 Japan; 7 Department of Hospital Pharmaceutics, School of Pharmacy, Showa University, 1-5-8, Hatanodai, Shinagawa-ku, Tokyo 142-8555 Japan

**Keywords:** Cost analysis, Colorectal cancer, Real-world setting, FOLFOX, FOLFIRI, XELOX, IRIS, Molecular targeted agents

## Abstract

**Background:**

Various chemotherapy regimens for advanced colorectal cancer have been introduced to clinical practice in Japan over the past decade. The cost profiles of these regimens, however, remain unclear in Japan. To explore the detailed costs of different regimens used to treat advanced colorectal cancer during the entire course of chemotherapy in patients treated in a practical setting, we conducted a so-called “real-world” cost analysis.

**Method:**

A detailed cost analysis was performed retrospectively. Patients with advanced colorectal cancer who had received chemotherapy in a practical healthcare setting from July 2004 through October 2010 were extracted from the ordering system database of Showa University Hospital. Direct medical costs of chemotherapy regimens were calculated from the hospital billing data of the patients. The analysis was conducted from a payer’s perspective.

**Results:**

A total of 30 patients with advanced colorectal cancer were identified. Twenty patients received up to second-line treatment, and 8 received up to third-line treatment. The regimens identified from among all courses of treatment in all patients were 13 oxaliplatin-based regimens, 31 irinotecan-based regimens, and 11 regimens including molecular targeted agents. The average (95 % confidence interval [95 % CI]) monthly cost during the overall period from the beginning of treatment to the end of treatment was 308,363 (258,792 to 357,933) Japanese yen (JPY). According to the type of regimen, the average monthly cost was 418,463 (357,413 to 479,513) JPY for oxaliplatin-based regimens, 215,499 (188,359 to 242,639) JPY for irinotecan-based regimens, and 705,460 (586,733 to 824,187) JPY for regimens including molecular targeted agents. Anticancer drug costs and hospital fees accounted for 50 to 77 % and 11 to 25 % of the overall costs of chemotherapy, respectively.

**Conclusion:**

The costs of irinotecan-based regimens were lower than those of oxaliplatin-based regimens and regimens including molecular targeted agents in Japan. Using a lower cost regimen for first-line treatment can potentially reduce the overall cost of chemotherapy. The main cost drivers were the anticancer drug costs and hospitalization costs.

**Electronic supplementary material:**

The online version of this article (doi:10.1186/s12913-015-1253-x) contains supplementary material, which is available to authorized users.

## Background

Colorectal cancer is currently one of the most common cancers worldwide [1]. In Japan the incidence of colorectal cancer was 119,000 in 2010 [2], and there were 48,000 deaths from the disease in 2013 [3]. The disease is already advanced at the time of diagnosis in 56 % of patients [2]. Surgery is the treatment of choice for early-stage colorectal cancer, while most patients with advanced colorectal cancer receive chemotherapy.

A combination of 5-fluorouracil (5-FU) and leucovorin (LV) was the standard regimen for advanced colorectal cancer during the 1980s and 1990s. After the year 2000, FOLFOX (oxaliplatin plus 5-FU and LV) and FOLFIRI (irinotecan plus 5-FU and LV) were developed and modified over the course of several years. These regimens extended progression-free survival to more than 8 months [4]. Furthermore, regimens including molecular targeted agents such as bevacizumab (Bev) or cetuximab (Cet) have prolonged progression-free survival to 9 to 11 months [5]. Currently, both FOLFOX and FOLFIRI with or without molecular targeted agents are commonly used to manage advanced colorectal cancer throughout the world [6–8]. Meanwhile, regimens including easy-to-administer oral agents were demanded, because both FOLFOX and FOLFIRI require continuous infusion of 5-FU for 2 days, which was not convenient in practice. A regimen combining the oral agent capecitabine with oxaliplatin (XELOX) was developed for the treatment of colorectal cancer. Two phase III trials showed that progression-free survival of patients who received XELOX is non-inferior to that of patients who received FOLFOX [9, 10], and XELOX is now used worldwide. In Japan, a regimen combining oral S-1 (a combination of tegafur, gimeracil, and oteracil), which was already widely used to treat gastric cancer, with irinotecan (IRIS) was developed for the management of advanced colorectal cancer [11]. The FIRIS study demonstrated that the progression-free survival of patients in the IRIS group was non-inferior to that in the FOLFIRI group [12], and IRIS is now one treatment option for this disease.

Despite the usefulness of various regimens for the treatment of advanced colorectal cancer, the development of new combination regimens has also attracted the attention of payers as well as physicians owing to the high costs of chemotherapy for colorectal cancer. The national medical expenditure in Japan reached 39.2 trillion JPY in 2011 [13]. Above all, the growing costs of cancer treatment are a burden to both the national medical expenditure and the out-of-pocket costs paid by patients. A number of researchers have reported the results of economic evaluations of colorectal cancer treatment in Japan [14–16]. All of these evaluations have provided useful information to decision makers and practitioners. However, the economic information in all except one [16] of these reports is insufficient for practical clinical use owing to the limited setting, such as clinical trials and controlled studies. In other words, cost-related data on the costs of chemotherapy in a practical clinical setting are thought to differ from data derived from planned clinical studies. Furthermore, the comprehensive economic profile of chemotherapy from first-line to the end of treatment for colorectal cancer is unclear in clinical trials. One task force of the International Society for Pharmacoeconomics and Outcomes Research (ISPOR) has reported the advantages of real-world data [17], and another task force of the ISPOR has provided a questionnaire to assess the relevance and credibility of observational studies designed to fill the gap between clinical trials and actual clinical practice [18].

We have recently reported the results of a cost-minimization analysis comparing S-1-based regimens with 5FU-based regimens for advanced colorectal cancer in Japan [19]. In the present study, we focused on the comprehensive economic profile of chemotherapy and performed a detailed cost analysis of various regimens used to manage this disease from first-line treatment to the end of chemotherapy in a real-world setting.

## Methods

### Analytical methods

We undertook a real-world cost analysis to compare the detailed costs of different chemotherapy regimens used for advanced colorectal cancer from first-line treatment to the end of chemotherapy in a practical setting. The analysis was retrospectively performed from a payer’s perspective. The costs of healthcare-related services were calculated from the payer side using billing data obtained from Showa University Hospital. Showa University Hospital is a general hospital located in Tokyo that has approximately 800 beds as well as outpatient clinics. The fees are the same for all payers and virtually all providers in Japan and are set by the government in the national fee schedule [20]. The study was approved by the Medical Ethics Committee of Showa University Hospital (approved number: 981).

We retrospectively collected clinical and claim data on patients with advanced colorectal cancer in this observational study. Because most of the patients died before this study began, it was difficult to obtain written consent from the patients. We analyzed the data after removing all personal information to protect privacy. This approach was in accordance with the Japanese Ethical Guidelines for Epidemiological Studies [21]; moreover, before the study began the Internal Review Board determined that consent is not required.

### Patients and chemotherapy regimens

The study group comprised patients who were given a diagnosis of advanced colorectal cancer and received chemotherapy until the end of the final line of chemotherapy in the Department of Surgery, Showa University Hospital during the period from July 2004 through October 2010. We extracted data on all lines of chemotherapy given to all patients and classified the regimens into the following three groups: oxaliplatin-based regimens, irinotecan-based regimens, and regimens including molecular targeted agents.

### Resource utilization data and costs

Resource utilization data on each chemotherapy regimen was extracted from Showa University Hospital billing data. The following costs were calculated on a monthly basis and were summed up on the basis of the Japanese National Health Insurance fee-for-service system (reimbursement price) in 2010: costs related to outpatient visits (physician consultations and outpatient visits); costs for hospitalization (basic bed charges, medical examinations, nursing care, and basic treatments during hospitalization); costs for operations and procedures (including transfusions); costs for laboratory tests and diagnostic imaging tests (radiography, computed tomography, magnetic resonance imaging, ultrasonography); costs for drugs and administration (anticancer drugs, antiemetics, and other drugs, including preparation and administration costs). Costs unrelated to cancer, such as costs required for the management of hypertension and hyperlipidemia, and operation fees, such as those required for the creation of an artificial anus, were not considered. Extra bed fees and meal fees were collected from billing data as a reference.

In this study, we collected data on regimens regardless of the treatment line, and then found that the duration of each regimen was influenced by the treatment line. The duration of the first line of treatment was longer than those of second and third lines. Therefore, the costs of each chemotherapy regimen were compared on a monthly basis to avoid the effects of the different durations of treatment.

### Time horizon and discounts

The monthly average costs of all chemotherapy regimens from first-line to the end of the final line of treatment were analyzed over the course of time. Treatments costs during the period of terminal care were not considered because it was difficult to collect relevant data during that period, and the costs varied depending on the status of individual patients during terminal care, regardless of the regimens used previously. Trends in the breakdown of costs were also analyzed in detail. Costs for more than 1 year were discounted by 3 %.

### Sensitivity analysis

The uncertainty of the results was explored by sensitivity analyses of uncertain factors. Several qualitative, one-way sensitivity analyses were conducted to explore the impact of alternative parametric assumptions on the results.

## Results

### Characteristics of patients and chemotherapy regimens

The characteristics of the 30 patients identified from the patient database of Showa University Hospital are shown in Table 1. The average age was 62.8 years. The major sites of colorectal cancer were the cecum in 4 patients, the ascending colon in 3, the transverse colon in 2, the descending colon in 1, the sigmoid colon in 7, and the rectum in 13. The rate of hospitalization was 23 %. The average duration of the entire course of chemotherapy was 13.5 months. Among the 30 patients, 20 received up to second-line chemotherapy, and 8 received up to third-line chemotherapy. The following different regimens were identified from among all courses of treatment in all patients: 13 oxaliplatin-based regimens (12 FOLFOX regimens and 1 XELOX regimen); 31 irinotecan-based regimens (27 IRIS regimens and 4 FOLFIRI regimens); 11 regimens including molecular targeted agents (6 FOLFOX + Bev regimens, 4 FOLFIRI + Bev or Cet regimens and 1 XELOX + Bev regimen); and 3 other regimens (2 Bev regimens and 1 Cet regimen). The schedules of each regimen are presented in Additional file 1.

**Table 1:** Patient characteristics

Number of patients	30
Sex	
Male	21
Female	9
Age	
−59	11
60–69	9
70−	10
Average	62.8
Site of colorectal cancer	
Cecum	4
Ascending colon	3
Transverse colon	2
Descending colon	1
Sigmoid colon	7
Rectum	13
Rate of hospitalization	23 %
Overall treatment period (average)	13.5 months
Treatment lines	
First-line	30
Second-line	20
Third-line	8
Regimens	
Oxaliplatin-based regimens	13
FOLFOX	12
XELOX	1
Irinotecan-based regimens	31
IRIS	27
FOLFIRI	4
Regimens including molecular targeted agents	11
FOLFOX + Bev	6
FOLFIRI + Bev or Cet	4
XELOX + Bev	1
Others	3

Overall treatment period = from the beginning of treatment to the end of the final line of chemotherapy (All of the patients had died or were in terminal care phase as of the study endpoint)

The numbers of each regimen were counted from among all courses of treatment in all patients

*FOLFOX* oxaliplatin plus 5-fluorouracil and leucovorin; *FOLFIRI* irinotecan plus 5-fluorouracil and leucovorin; *XELOX* oxaliplatin plus capecitabine; *IRIS* irinotecan plus S-1 (tegafur, gimeracil and oteracil combination); *Bev* bevacizumab; *Cet* cetuximab; *Others* 2 Bev and 1 Cet

The numbers of regimens and the durations of each treatment line are shown in Table 2. Two oxaliplatin-based regimens (2 FOLFOX regimens), 27 irinotecan-based regimens (27 IRIS regimens), and 1 regimen including molecular targeted agents (1 FOLFIRI + Bev regimen) were given as first-line treatment. Eleven oxaliplatin-based regimens (10 FOLFOX regimens, 1 XELOX regimen), 8 regimens including molecular targeted agents (6 FOLFOX + Bev regimens, 1 FOLFIRI + Cet regimen, 1 XELOX + Bev regimen) and 1 Bev regimen were given as second-line treatment. Four irinotecan-based regimens (4 FOLFIRI regimens), 2 regimens including molecular targeted agents (1 FOLFIRI + Bev regimen, 1 FOLFIRI + Cet regimen), 1 Bev regimen and 1 Cet regimen were given as third-line treatment. The average duration of each line was 9.0 months for first-line, 3.9 months for second-line, and 3.8 months for third-line. The average duration of chemotherapy according to the type of regimen was 3.9 months for oxaliplatin-based regimens, 8.8 months for irinotecan-based regimens, and 4.3 months for regimens including molecular targeted agents.

**Table 2:** Numbers of regimens used in each treatment line and durations of each treatment line

	First-line	Second-line	Third-line	Average durations of treatment (month)
Total	30	20	8	6.5
Oxaliplatin-based regimens	2	11		3.9
FOLFOX	2	10		3.8
XELOX		1		5.0
Irinotecan-based regimens	27		4	8.8
IRIS	27			9.3
FOLFIRI			4	5.8
Regimens including molecular targeted agents	1	8	2	4.3
FOLFOX + Bev		6		5.0
FOLFIRI + Bev or Cet	1	1	2	4.0
XELOX + Bev		1		1.0
Others		1	2	1.3
Average durations of treatment (month)	9.0	3.9	3.8	

### Costs

#### Total costs for the entire course of chemotherapy and costs according to regimen group

**Fig. 1:**
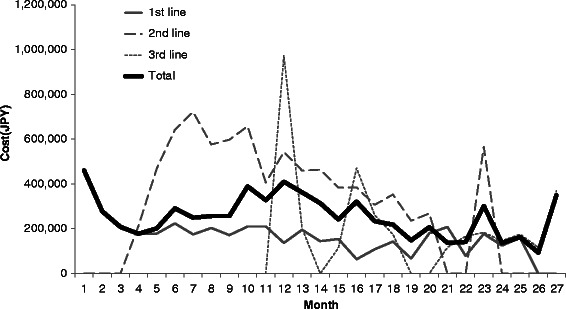
Monthly total costs from the beginning of treatment to the end of treatment. The costs of each line and costs from the beginning of treatment to the end of treatment are shown. The earliest starting point of second-line treatment was the fourth month and that of third-line treatment was the twelfth month

**Fig. 2:**
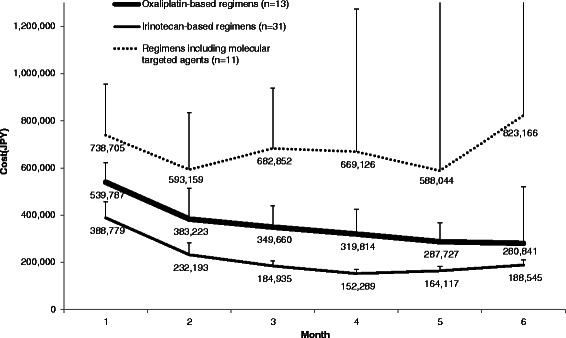
Total costs of each regimen up to the sixth month (mean + 95 % CI)

The monthly total costs are shown in Table 3 and Fig. 1, and the time courses of the total costs of each regimen group are shown in Fig. 2.

**Table 3:** Average monthly total costs (JPY)

	Total costs (mean)	95 % CI	Minimum	25th percentile	Median	75th percentile	Maximum
Overall treatment period (*n* = 30)	308,363	(258,792–357,933)	152,345	208,653	270,328	340,179	637,299
First-line treatment (*n* = 30)	256,149	(207,992–304,307)	149,816	186,293	208,555	261,922	637,299
Second-line treatment (*n* = 20)	518,897	(431,290–606,504)	280,977	348,454	431,758	650,541	925,339
Third-line treatment (*n* = 8)	416,251	(188,316–644,185)	141,582	162,033	297,117	600,389	1,056,666
Oxaliplatin-based regimens (*n* = 13)	418,463	(357,413–479,513)	280,977	339,667	399,767	444,406	635,115
Irinotecan-based regimens (*n* = 31)	215,499	(188,359–242,639)	141,867	171,990	207,516	223,415	567,549
Regimens including molecular targeted agents (*n* = 11)	705,460	(586,733–824,187)	411,384	597,586	710,434	822,972	1,056,666

Costs during the overall treatment period were calculated from the beginning of treatment to the end of treatment and were discounted by 3 % after the twelfth month

The costs of first-line, second-line, and third-line treatments were calculated until the end of each treatment and were discounted by 3 % after the twelfth month

The costs of each regimen group were calculated until the twelfth month and were not discounted

The average (95 % CI, minimum, 75th percentile, median, 75th percentile, maximum) monthly cost during the overall period from the beginning of treatment to the end of treatment was 308,363 (258,792 to 357,933, 152,345, 208,653, 270,328, 340,179, 637,299) JPY. The monthly costs varied over time. The first cost peak occurred in the first month, and the cost gradually increased from the fifth to the twelfth month. During this period, some patients started to receive expensive second-line treatments. The second cost peak occurred in the twelfth month and overlapped with the peak in the first month of third-line treatment. The average (95 % CI, minimum, 75th percentile, median, 75th percentile, maximum) monthly costs were 256,149 (207,992 to 304,307, 149,816, 186,293, 208,555, 261,922, 637,299) JPY for first-line treatment, 518,897 (431,290 to 606,504, 280,977, 348,454, 431,758, 650,541, 925,339) JPY for second-line, and 416,251 (188,316 to 644,185, 141,582, 162,033, 297,117, 600,389, 1,056,666) JPY for third-line.

The average (95 % CI, minimum, 75th percentile, median, 75th percentile, maximum) monthly costs of oxaliplatin-based regimens, irinotecan-based regimens, and regimens including molecular targeted agents were respectively 418,463 (357,413 to 479,513, 280,977, 339,667, 399,767, 444,406, 635,115) JPY, 215,499 (188,359 to 242,639, 141,867, 171,990, 207,516, 223,415, 567,549) JPY, and 705,460 (586,733 to 824,187, 411,384, 597,586, 710,434, 822,972, 1,056,666) JPY. The costs of irinotecan-based regimens were lower than those of oxaliplatin-based regimens and regimens including molecular targeted agents.

#### Cost-component analysis

The monthly component costs of each regimen group up to the twelfth month of treatment are shown in Table 4.

**Table 4:** Average monthly component costs of each regimen group up to the twelfth month (JPY)

	Oxaliplatin-based regimens	Irinotecan-based regimens	Regimens including molecular targeted agents
	(*n* = 13)	(*n* = 31)	(*n* = 11)
	mean (range)	mean (range)	mean (range)
Total	418,463 (280,977–635,115)	215,499 (141,867–567,549)	705,460 (411,384–1,056,666)
Outpatient visits	6,051 (500–11,000)	6,329 (3,540–11,000)	5,375 (0–10,488)
Hospitalization	89,935 (14,022–209,077)	53,584 (0–375,985)	77,752 (0–192,950)
Operations and Procedures	37,353 (0–178,780)	455 (0–9,860)	38,222 (0–178,273)
Tests			
Laboratory tests	15,289 (4,991–38,970)	11,757 (5,548–41,200)	12,448 (5,833–24,173)
Diagnostic imaging tests	8,512 (0–29,140)	24,039 (1,493–56,330)	8,958 (0–23,118)
Drugs and Administration			
Anticancer drugs	228,539 (136,433–364,332)	96,853 (58,335–138,086)	535,214 (177,458–988,262)
Antiemetics	8,688 (6,051–19,636)	9,232 (5,857–12,557)	6,746 (365–11,972)
Other drugs	3,707 (0–19,128)	708 (0–4,347)	4,546 (0–32,316)
Administration	19,882 (2,200–44,160)	12,543 (950–42,930)	16,133 (950–43,610)
Others	508 (0–6,600)	0	67(0–733)
Proportion of hospitalization	21 %	25 %	11 %
Proportion of anticancer drugs	58 %	50 %	77 %
Meal fees	8,677	4,001	4,639
Extra bed fees	67,290	13,364	10,350

Outpatient visits = physician consultations and outpatient visits; Hospitalization = basic bed charges, medical examinations, nursing care, and basic treatments during hospitalization; Operations = including transfusion; Diagnostic imaging tests = X-ray, CT, MRI, ultrasound; Administration = preparation and management;

Range = minimum to maximum

The cost drivers were anticancer drugs and hospitalization. The costs of anticancer drugs were 228,539 JPY (proportion of total: 58 %) for oxaliplatin-based regimens, 96,853 JPY (50 %) for irinotecan-based regimens, and 535,214 JPY (77 %) for regimens including molecular targeted agents. The costs were lower for irinotecan-based regimens than for oxaliplatin-based regimens and regimens including molecular targeted agents. The hospital fees were 89,935 JPY (21 %) for oxaliplatin-based regimens, 53,584 JPY (25 %) for irinotecan-based regimens, and 77,752 JPY (11 %) for regimens including molecular targeted agents. Next to the costs of anticancer drugs, hospital fees were second highest costs for all regimen groups.

The costs of other components were as follows. The administration costs for drug administration were 19,882 JPY for oxaliplatin-based regimens and 16,133 JPY for regimens including molecular targeted agents, which were higher than the 12,543 JPY for irinotecan-based regimens. The diagnostic imaging cost for irinotecan-based regimens was 24,039 JPY, which was higher than the costs in the other regimen groups. The operation fees were 37,353 JPY for oxaliplatin-based regimens and 38,222 JPY for regimens including molecular targeted agents, which were higher than the operation fees for irinotecan-based regimens.

### Sensitivity analysis

The results of sensitivity analysis are shown in Table 5. As mentioned above, the treatment line differed among the regimens in this study. There was some bias, which most likely depended on the treatment line. Regimens used as first-line during the first month of treatment tended to be administered in the hospital, and the hospital fee accounted for a large proportion of total costs and might have influenced the results. We therefore initially performed a simple sensitivity analysis to examine the hospital fee. Two scenarios were considered: 3 days’ hospitalization and an outpatient setting. As a result, the orders of the total costs among the regimen groups given on an inpatient basis and an outpatient basis were basically same as the order of the total costs in the regimen groups for the base-case. As for oxaliplatin-based regimens and regimens including molecular targeted agents, the base-case costs were slightly higher than those of a 3-day hospitalization setting because the period of hospitalization was longer than 3 days for the base-case. The costs for diagnostic imaging tests were higher for irinotecan-based regimens than for the other regimen groups because IRIS, which was the most commonly used irinotecan-based regimen, was mainly used for first-line treatment, during which diagnostic imaging tests were frequently performed to assess disease progression. Therefore, we performed a simple sensitivity analysis of the costs for diagnostic imaging tests. The results demonstrated that the cost of diagnostic imaging tests did not have an impact on the total costs. In addition, because the use of generic anticancer drugs may have influenced the results, we performed a simple sensitivity analysis comparing the use of generic leucovorin with that of brand leucovorin. The results did not change.

**Table 5:** Sensitivity analysis (average monthly total costs: JPY)

	Oxaliplatin-based regimens	Irinotecan-based regimens	Regimens including molecular targeted agents
	(*n* = 13)	(*n* = 31)	(*n* = 11)
Base-case	418,463	215,499	705,460
Hospitalization	390,487	223,597	690,343
Outpatient	327,177	160,287	627,033
Imaging Max	433,991	215,499	720,541
Imaging Min	418,463	199,972	705,013
Brand drug	418,463	215,888	707,715
Generic drug	404,097	214,046	694,883

Hospitalization = in the scenario of 3 days’ hospitalization; Outpatient = in the scenario of outpatient visit; Imaging Max = in the scenario of maximum utilization of diagnostic imaging tests; Imaging Min = in the scenario of minimum utilization of diagnostic imaging tests; Brand drug = in the scenario of using brand leucovorin; Generic drug = in the scenario of using generic leucovorin

## Discussion

Current clinical guidelines for colorectal cancer [6–8], recommend FOLFOX or FOLFIRI with or without molecular targeted agent such as Bev or Cet as first-line treatment. In the present study, however, the main regimen used for first-line treatment was the oral S-1-based IRIS regimen. IRIS was approved for reimbursement for colorectal cancer in 2003 under Japanese National Health Insurance before the present study began, and the FIRIS study comparing IRIS with FOLFIRI started in 2006 [12]. Therefore, physicians used the more convenient oral IRIS regimen as first-line chemotherapy during this study, because they expected IRIS to be as effective as FOLFIRI, one of the standard regimens used to treat advanced colorectal cancer. On the other hand, molecular targeted agents and XELOX were virtually not used as first-line treatment during this study, because these treatments were approved for reimbursement under Japanese National Health Insurance in 2007 and 2009, respectively, which was during the latter part of this study.

In the present study, the average duration of the entire course of chemotherapy was longer than 1 year, and the average monthly total cost during the entire period was 308,363 JPY, which nearly equals the average wage in Japan [22]. The burden to health insurance and individuals was thus substantial. The duration of first-line treatment was 9.0 months, which was longer than the duration of second and subsequent lines of chemotherapy. Therefore, the cost of the entire course of chemotherapy seemed to depend primarily on the regimen used for first-line treatment. In this study, inexpensive IRIS was used for first-line treatment, which contributed to a lower overall cost for chemotherapy.

The costs of oxaliplatin-based regimens such as FOLFOX were higher than those of irinotecan-based regimens such as IRIS and FOLFIRI. Regimens including molecular targeted agents such as Bev and Cet were the most expensive. Many economic evaluations of colorectal cancer chemotherapy have been performed in the world (Table 6). Miyazaki et al., Shiroiwa et al., and Ajima et al. have reported on the costs of chemotherapy for advanced colorectal cancer in Japan [14–16]. All of these studies showed that the cost of FOLFOX is higher than that of FOLFIRI, consistent with the results of our study. In the United States, several economic evaluations of the FOLFOX and FOLFIRI regimens have also reported that FOLFOX is more expensive than FOLFIRI [23, 24]. In the United Kingdom, however, the costs of FOLFOX and FOLFIRI are about the same [25, 26]. These differences seem to be attributed to the different drug prices in each country. As for molecular targeted agents, several economic evaluations of Bev have been reported by Shiroiwa et al., Tappenden et al., and Lee et al. [15, 27, 28]. These studies showed that the cost-effectiveness of Bev is low. In the United Kingdom, the National Institute for Health and Care Excellence (NICE) did not recommend molecular targeted agents as first-line treatment for advanced colorectal cancer [29].

**Table 6:** Costs of chemotherapy for advanced colorectal cancer in the world (USD, monthly)

	Oxaliplatin-based regimens	Irinotecan-based regimens	Regimens including molecular targeted agents
Japan (Present study)	FOLFOX: 4,141	IRIS: 2,217	FOLFOX + Bev: 7,353
		FOLFIRI (2^nd^ line): 1,574	FOLFIRI + Bev or Cet: 6,777
Japan (Miyazaki^14^)	FOLFOX: 4,151	FOLFIRI: 2,398	
Japan (Shiroiwa^15^)	FOLFOX: 4,554	FOLFIRI (2^nd^ line): 2,376	FOLFOX + Bev: 7,722
	XELOX: 3,564		IFL + Bev: 4,950
			XELOX + Bev: 6,831
Japan (Ajima^16^)	FOLFOX: 5,352	FOLFIRI: 3,743	
US (Mullins^23^)	FOLFOX: 60,179 (per patient)	FOLFIRI: 44,087 (per patient)	
US (Tumeh^24^)	FOLFOX: 29,865 (per patient)	FOLFIRI: 24,551 (per patient)	
US (Chu^30^)	FOLFOX: 14,300		
	XELOX: 11,473		
UK (NICE:HTA2001^25^)	FOLFOX: 5,718	FOLFIRI: 5,975	
UK (NICE:HTA2008^26^)	FOLFOX: 22,734 (per patient)	FOLFIRI: 23,017 (per patient)	
UK (Tappenden^27^)		IFL: 40,998 (per patient)	IFL + Bev: 74,379 (per patient)
France (Perrocheau^32^)	FOLFOX: 23,597 (per patient)		
	XELOX: 17,695 (per patient)		
Koria (Lee^28^)		FOLFIRI: 1,597	Bev: 5,150
UK (Ward^31^)	5-FU/LV (Mayo): 2,065		
	Capecitabine: 1,225		
	UFT/LV: 1,944		

*IFL* irinotecan plus fluorouracil and leucovorin; *5-FU* 5-fluorouracil; *LV* leucovorin; *UFT* tegafur and uracil combination

Rate: 1 USD (US Dollar) = 101 JPY, 0.58 GBP (British Pound), 0.73 EUR (Euro) (2014/7/9)

Hospital fees were thought to be one of the cost drivers. However, the results of sensitivity analysis showed that the hospital fees did not influence the total costs. The base-case costs of oxaliplatin-based regimens and regimens including molecular targeted agents were higher than the cost of the scenario assuming 3 days of hospitalization. The management of adverse effects was apparently more difficult for oxaliplatin-based regimens and regimens including molecular targeted agents than for other regimens and thus required a longer hospitalization period.

The cost characteristics of other components were as follows. The costs associated with drug administration were higher for oxaliplatin-based regimens (FOLFOX, etc.) than for irinotecan-based regimens (IRIS, etc.) because IRIS regimens did not require continuous infusion of 5-FU. The diagnostic imaging costs for irinotecan-based regimens, used mainly as first-line treatment, were higher than those for other regimens because diagnostic imaging was frequently performed during first-line treatment. Operation fees for placement of a central venous access port for continuous infusion of 5-FU were high for oxaliplatin-based regimens, because most FOLFOX regimens were used before FOLFIRI in this study. The costs of antiemetics did not differ among the regimens.

As reference information, we also collected data on the extra bed fee paid by patients, which was not included in total costs in this study. The price depended on the type of room, irrespective of the type of regimen. Such out-of-pocket costs paid by the patient, however, were a great burden in a practical setting. We need to consider such out-of-pocket costs from a societal perspective in future studies.

Our analysis had several important limitations. First, a small sample of claim data was collected from a single institution in this study. It seems to be difficult to generalize the results of this study. However, standard dosages and schedules of the regimens in Showa University Hospital were used. In addition, by grouping the regimens to compensate for the small numbers of patients who received each regimen, we could obtain convincing results to some extent. Second, the treatment line differed among the regimens. However, we did not attempt to balance the background characteristics of the patients according to each regimen, because treatment dosages and schedules did not differ between the lines. With regard to this point, the durations and total costs of each regimen group varied according to the treatment line. Therefore, we compared the regimen groups on a monthly basis to eliminate potential effects related to differences in the duration of each treatment. Ideally, however, we have to directly compare total costs in a clinical trial. Third, we calculated only direct costs because our analysis was performed from a payer’s perspective.

As mentioned above, the most widely used regimen in this study was oral S-1-based IRIS. Several economic evaluations of regimens including oral preparations such as capecitabine and tegafur-uracil have shown that such regimens are more cost effective than regimens comprising only injectable preparations [15, 30–32]. In our former study analyzing treatment costs for gastric cancer [33], S-1 regimens were also less expensive and more convenient than injectable regimens. Furthermore, in our other study of colorectal cancer in an outpatient setting [19], S-1-based regimens were cost saving as compared with 5-FU-based regimens. This result was derived from the analysis including time costs of administration of anticancer drugs. Therefore, our findings suggest that further cost-effectiveness analyses comparing the IRIS regimen with other regimens, taking into account societal costs, time-related costs, and quality of life, are warranted. We hope that our results will help decision makers obtain a more comprehensive view of costs and thereby narrow the gap between clinical trials and actual clinical practice and will help clinicians select regimens for individual patients.

## Conclusions

The cost of the entire course of chemotherapy for advanced colorectal cancer was considerably expensive in Japan. The costs of irinotecan-based regimens such as IRIS and FOLFIRI were lower than those of oxaliplatin-based regimens, such as FOLFOX, and regimens including molecular targeted agents, such as FOLFOX + Bev and FOLFIRI + Bev or Cet. Using lower cost regimens such as IRIS as first-line chemotherapy can lead to a reduction in overall costs for the entire course of chemotherapy, because the duration of first-line treatment was longer than the duration of second and subsequent lines of chemotherapy. The main cost drivers of treatment for advanced colorectal cancer were anticancer drug costs and hospital expenses.

## Additional file

Additional file 1: **Schedule of each regimen.** (DOCX 15 kb)

## Abbreviations

FOLFOX: oxaliplatin plus 5-fluorouracil and leucovorin
FOLFIRI: irinotecan plus 5-fluorouracil and leucovorin
XELOX: oxaliplatin plus capecitabine
IRIS: irinotecan plus S-1
IFL: irinotecan plus 5-fluorouracil and leucovorin
5-FU: 5-fluorouracil
LV: leucovorin
S-1: tegafur, gimeracil, and oteracil combination
UFT: tegafur and uracil combination
Bev: bevacizumab
Cet: cetuximab
