# Supplementary material for: Real-world cost analysis of chemotherapy for colorectal cancer in Japan: detailed costs of various regimens during the entire course of chemotherapy
Source: BMC Health Serv Res. 2016 Jan 4;16:2. doi: 10.1186/s12913-015-1253-x (PMC4698819; doi:10.1186/s12913-015-1253-x)
Supplement: Additional file 1: — Schedule of each regimen. (DOCX 15 kb) [file 12913_2015_1253_MOESM1_ESM.docx]

Additional file 1

Schedule of each regimen

| FOLFOX | oxaliplatin 85 mg/m^2^, l-leucovorin 200 mg/m^2^, and 5-fluorouracil bolus 400 mg/m^2^ on day 1 and a continuous infusion of 5-fluorouracil 2400 mg/m^2^ over the course of 46 h every 2 weeks |
| --- | --- |
| XELOX | oxaliplatin 130 mg/m^2^ on day 1 and capecitabine 1000 mg/m^2^ twice daily for 14 days every 3 weeks |
| IRIS | irinotecan 80 mg/m^2^ on days 1 and 15 and S-1 40-60 mg twice daily for 21 days every 5 weeks |
| FOLFIRI | irinotecan 180 mg/m^2^, l-leucovorin 200 mg/m^2^, and 5-fluorouracil bolus 400 mg/m^2^ on day 1 and a continuous infusion of 5-fluorouracil 2400 mg/m^2^ over the course of 46 h every 2 weeks |
| FOLFOX+Bev | FOLFOX plus bevacizumab 5 mg/kg on day 1 |
| FOLFIRI+Bev or Cet | FOLFIRI plus bevacizumab 5 mg/kg or cetuximab 250-400 mg/m^2^ on day 1 |
| XELOX+Bev | XELOX plus bevacizumab 7.5 mg/kg on day 1 |
